# Supplementary material for: We shape our buildings, but do they then shape us? A longitudinal analysis of pedestrian flows and development activity in Melbourne
Source: PLoS One. 2021 Sep 21;16(9):e0257534. doi: 10.1371/journal.pone.0257534 (PMC8454953; doi:10.1371/journal.pone.0257534)
Supplement: S1 File — (DOCX) [file pone.0257534.s002.docx]

**S1 File**

**S1 Note.** Accounting for trip elasticity.

In addition to using the Huff probability model to allocate trips to competing destinations k, we include a Gravity Accessibility term, which ensures that the number of trips from [j] to [k] also depend on the weights and proximities of destinations k:

$$Trips\left[ j,k \right]=W\left[ j \right]\cdot Probability\left[ j,k \right]\cdot\frac{{W[k]}^{\alpha}}{e^{\beta\cdot d\left[ j,k \right]}}$$

Consider for example a single trip origin (building) with 10 people in it, and a single destination within its walking radius, such as a restaurant with a size of 1,000 sqft. Depending on how far and big the destination is, we find the rate of trips sent to this destination. Now, if another business establishment is added to the destination building (adding a weight of 1,000 sqft), then that building obtains a new (larger) weight of 2,000sqft. This would result in allocating more trips from the same origin, even when origin weight stays the same at 10 people. Our elasticity effect ensures that when destination weights expand, or when they are closer to the origin, trip generation increases.

An alternative approach for modeling trip generation elasticity with respect to destination availability is to adjust origin weights a priori based on destination accessibility for each kind of trip. For office to F&B trips, for instance, we can first measure how readily each office location can access surrounding F&B establishments on foot (using the Gravity Accessibility index for instance), and then adjust the trip generation weights at the said origins accordingly.

**S1 Table.** Bivariate correlations between estimated flows and sensor counts for AM, Lunch and PM periods on different years. Sensor counts indicate average recorded counts across all workdays in June in a given year.

|  | **AM Peak** | 2014 | 2015 | 2016 | 2017 | 2018 | 2019 |
| --- | --- | --- | --- | --- | --- | --- | --- |
| 1 | Employment – Train station | 0.79 | 0.71 | 0.67 | 0.45 | 0.54 | 0.60 |
| 2 | Employment – Tram stop | 0.40 | 0.32 | 0.33 | 0.14 | 0.23 | 0.17 |
| 3 | Employment – F&B | -0.07 | -0.11 | -0.07 | -0.07 | 0.00 | -0.03 |
| 4 | Employment – Park | 0.54 | 0.42 | 0.41 | 0.05 | -0.05 | -0.09 |
| 5 | Employment – Parking | 0.25 | 0.11 | 0.11 | 0.04 | 0.09 | 0.04 |
| 6 | Employment – Residential | 0.18 | 0.06 | 0.18 | 0.08 | 0.17 | 0.17 |
| 7 | Residential – Tram stop | 0.38 | 0.21 | 0.45 | 0.37 | 0.34 | 0.09 |
| 8 | Residential – Park | 0.50 | 0.29 | 0.38 | 0.10 | -0.05 | -0.13 |
| 9 | Amenities – Amenities | -0.02 | -0.11 | -0.08 | -0.03 | -0.03 | -0.08 |
| 10 | Tram stop – Tourist site | -0.10 | -0.05 | -0.01 | 0.00 | 0.02 | -0.03 |
|  |  |  |  |  |  |  |  |
|  | **Lunch** | 2014 | 2015 | 2016 | 2017 | 2018 | 2019 |
| 1 | Employment – Train station | 0.13 | 0.40 | 0.36 | 0.26 | 0.45 | 0.42 |
| 2 | Employment – Tram stop | 0.40 | 0.35 | 0.38 | 0.30 | 0.35 | 0.32 |
| 3 | Employment – F&B | 0.30 | 0.20 | 0.22 | 0.19 | 0.23 | 0.18 |
| 4 | Employment – Park | -0.14 | -0.08 | -0.10 | -0.20 | -0.23 | -0.24 |
| 5 | Employment – Parking | 0.07 | 0.03 | 0.03 | -0.05 | 0.02 | -0.04 |
| 6 | Employment – Residential | 0.25 | 0.13 | 0.17 | 0.10 | 0.11 | 0.09 |
| 7 | Residential – Tram stop | 0.31 | 0.15 | 0.19 | 0.28 | 0.20 | 0.04 |
| 8 | Residential – Park | -0.09 | -0.12 | -0.14 | -0.15 | -0.27 | -0.28 |
| 9 | Amenities – Amenities | 0.61 | 0.45 | 0.48 | 0.44 | 0.44 | 0.45 |
| 10 | Tram stop – Tourist site | -0.05 | -0.02 | 0.04 | 0.02 | 0.03 | 0.03 |
|  |  |  |  |  |  |  |  |
|  | **PM Peak** | 2014 | 2015 | 2016 | 2017 | 2018 | 2019 |
| 1 | Employment – Train station | 0.47 | 0.60 | 0.56 | 0.35 | 0.52 | 0.53 |
| 2 | Employment – Tram stop | 0.45 | 0.37 | 0.39 | 0.25 | 0.32 | 0.25 |
| 3 | Employment – F&B | 0.21 | 0.07 | 0.11 | 0.10 | 0.16 | 0.09 |
| 4 | Employment – Park | 0.10 | 0.13 | 0.11 | -0.15 | -0.20 | -0.21 |
| 5 | Employment – Parking | 0.17 | 0.05 | 0.05 | -0.01 | 0.04 | -0.02 |
| 6 | Employment – Residential | 0.25 | 0.09 | 0.18 | 0.10 | 0.13 | 0.11 |
| 7 | Residential – Tram stop | 0.40 | 0.20 | 0.35 | 0.33 | 0.26 | 0.04 |
| 8 | Residential – Park | 0.24 | 0.11 | 0.13 | -0.01 | -0.18 | -0.21 |
| 9 | Amenities – Amenities | 0.26 | 0.17 | 0.18 | 0.16 | 0.20 | 0.15 |
| 10 | Tram stop – Tourist site | -0.12 | -0.04 | 0.00 | 0.00 | 0.02 | -0.01 |
|  |  |  |  |  |  |  |  |
|  | Note: | >0.05 | >0.1 |  |  |  |  |

**S1 Fig.** Left**:** Weekend/Weekday Activity Variation. Right: Mean pedestrian counts on dry versus rainy days in 2018. Each infrared sensor counts pedestrian traffic that traverses through a virtual gate across a sidewalk. Sensors only count footfall on one side of a street. Data aggregated to an hourly count is currently available 24 hours a day from 62 sensors. In the period of observation from January 2014 through January 2019, 25 sensors were active throughout, while some were deactivated and others added over the years. Observed pedestrian counts (Fig 6) showed the lightest foot traffic in the AM peak (mean count among 25 sensors=903 pax/hr), higher during the lunch period (mean=1,309 pax/hr) and highest during the PM peak (mean=1,541 pax/hr). Counts trend upward in the years studied, in accord with Melbourne’s growing population and employment numbers.


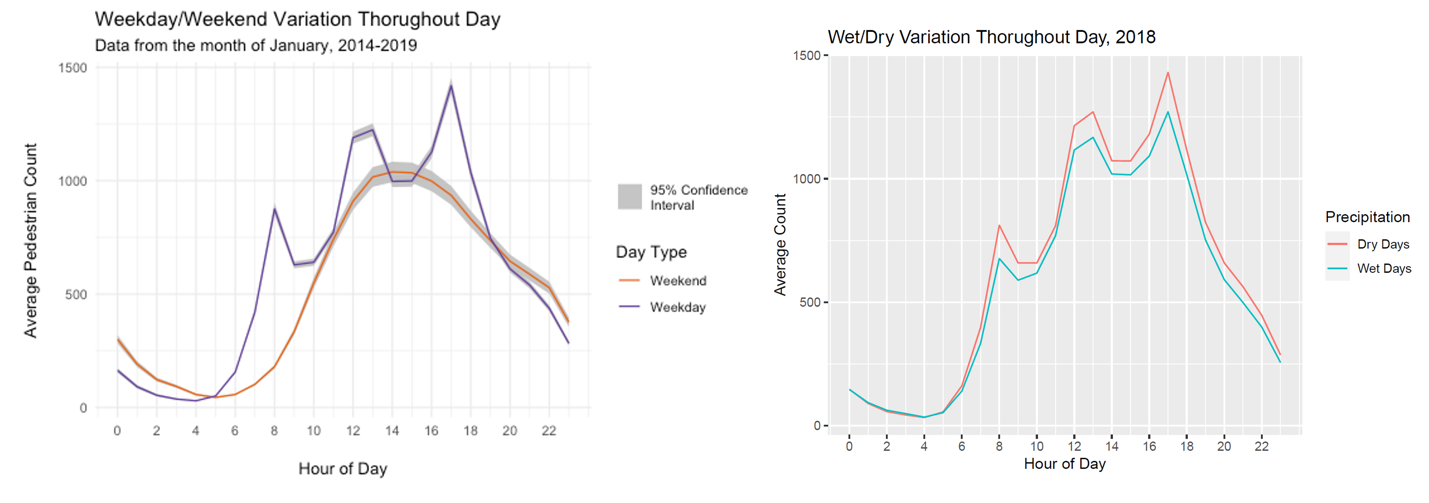


**S2 Fig**. Partial Dependence plots of 2014 predictive variables. Non-linear relationships are shown between pedestrian flow and some of the predictors.


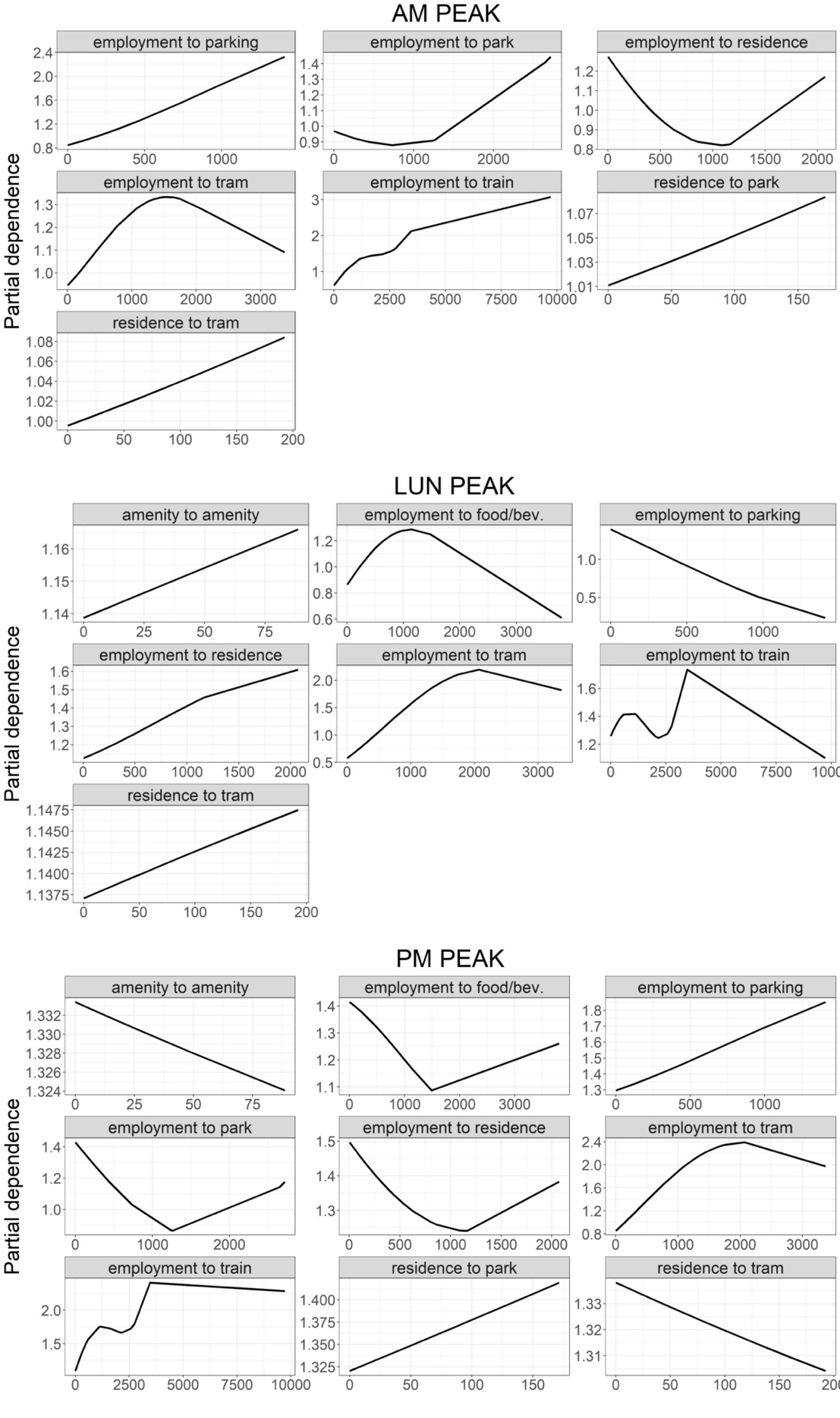


**S2 Table.** Goodness of fit results of 10 types of pedestrian flows combined with weather and day-of-week variables, without sensor-level dummies. Top: calibration results on June 2014 training data. Bottom: prediction results on June 2015 data.

| **Calibration on June 2014 training data (Mon-Fri)** | | | | | |  |  |  |
| --- | --- | --- | --- | --- | --- | --- | --- | --- |
| **Peak** | **Metric** | **MLM** | **SGD** | **SVR** | **RF** | **BAG** | **GB** | **GP** |
| AM | R2 | 0.65 | 0.72 | 0.89 | 0.99 | 0.9 | 0.99 | 0.72 |
|  | MAE | 0.47 | 0.41 | 0.22 | 0.05 | 0.2 | 0.07 | 0.42 |
|  | RMSE | 0.66 | 0.58 | 0.36 | 0.13 | 0.35 | 0.12 | 0.58 |
| LUNCH | R2 | 0.55 | 0.47 | 0.88 | 0.99 | 0.88 | 0.98 | 0.47 |
|  | MAE | 0.49 | 0.54 | 0.22 | 0.04 | 0.2 | 0.09 | 0.54 |
|  | RMSE | 0.62 | 0.67 | 0.32 | 0.07 | 0.32 | 0.14 | 0.67 |
| PM | R2 | 0.43 | 0.56 | 0.89 | 0.99 | 0.89 | 0.96 | 0.57 |
|  | MAE | 0.65 | 0.55 | 0.24 | 0.05 | 0.23 | 0.11 | 0.54 |
|  | RMSE | 0.79 | 0.69 | 0.35 | 0.1 | 0.35 | 0.2 | 0.68 |
|  |  |  |  |  |  |  |  |  |
| **Prediction on June 2015 data (Mon-Fri)** | | | | |  |  |  |  |
| **Peak** | **Metric** | **MLM** | **SGD** | **SVR** | **RF** | **BAG** | **GB** | **GP** |
| AM | R2 | 0.48 | 0.53 | 0.53 | 0.48 | 0.53 | 0.39 | 0.52 |
|  | MAE | 0.53 | 0.56 | 0.51 | 0.42 | 0.52 | 0.5 | 0.57 |
|  | RMSE | 0.81 | 0.8 | 0.8 | 0.84 | 0.81 | 0.91 | 0.81 |
| LUNCH | R2 | 0.33 | 0.28 | 0.57 | 0.28 | 0.56 | 0.33 | 0.28 |
|  | MAE | 0.62 | 0.66 | 0.46 | 0.5 | 0.47 | 0.5 | 0.66 |
|  | RMSE | 0.8 | 0.9 | 0.7 | 0.91 | 0.71 | 0.87 | 0.91 |
| PM | R2 | 0.29 | 0.45 | 0.55 | 0.29 | 0.53 | 0.37 | 0.44 |
|  | MAE | 0.81 | 0.77 | 0.59 | 0.71 | 0.61 | 0.68 | 0.76 |
|  | RMSE | 1.05 | 1.0 | 0.9 | 1.13 | 0.92 | 1.07 | 1.01 |

**S3 Table.** Multi-level model with random intercepts, fixed slopes, and random effects for sensors and day-of-week. Standardized coefficients are presented for the models calibrated on June 2014 data.

| **Multi-level model estimation results (June 2014 calibration)** | | | | | | |
| --- | --- | --- | --- | --- | --- | --- |
|  | | | | | | |
|  | *Dependent variable:* | | | | | |
|  |  | | | | | |
|  | **AM Peak** | | **Lunch Peak** | | **PM Peak** | |
|  | ***All effects*** | ***Positive effects only*** | ***All effects*** | ***Positive effects only*** | ***All effects*** | ***Positive effects only*** |
|  | (1) | (2) | (3) | (4) | (5) | (6) |
|  | | | | | | |
| Constant | 696.271^***^ | 697.123^***^ | 1,182.265^***^ | 1,180.735^***^ | 1,445.887^***^ | 1,444.726^***^ |
|  | p = 0.00000 | p = 0.00001 | p = 0.000 | p = 0.000 | p = 0.000 | p = 0.000 |
| Employment – Train station | 892.600^***^ | 734.025^***^ | 223.859 |  | 675.130^***^ | 353.619^**^ |
|  | p = 0.000 | p = 0.000 | p = 0.103 |  | p = 0.00001 | p = 0.029 |
| Employment – Tram stop | 239.731 |  | 264.741 |  | 662.715^**^ | 75.603 |
|  | p = 0.343 |  | p = 0.301 |  | p = 0.014 | p = 0.711 |
| Employment – F&B | -406.333^**^ |  | 10.169 |  | -180.407 |  |
|  | p = 0.017 |  | p = 0.953 |  | p = 0.314 |  |
| Employment – Park | -481.067^***^ |  | -393.440^***^ |  | -885.529^***^ |  |
|  | p = 0.002 |  | p = 0.010 |  | p = 0.00000 |  |
| Employment – Parking | 621.689^***^ | 215.720^*^ | -43.535 |  | 242.167 |  |
|  | p = 0.0005 | p = 0.089 | p = 0.809 |  | p = 0.198 |  |
| Residential – Tram stop | 111.307 | 46.334 | 237.186^*^ | 75.844 | 126.634 | 106.466 |
|  | p = 0.401 | p = 0.724 | p = 0.077 | p = 0.490 | p = 0.367 | p = 0.555 |
| Residential- Employment | -353.799^***^ |  | -443.955^***^ |  | -595.009^***^ |  |
|  | p = 0.005 |  | p = 0.0004 |  | p = 0.00001 |  |
| Residential – Park | 289.584^***^ | 177.634 | 124.107 | 73.266 | 383.903^***^ | 147.288 |
|  | p = 0.006 | p = 0.125 | p = 0.235 | p = 0.494 | p = 0.0005 | p = 0.337 |
| Amenities – Amenities | 0.071 |  | 568.003^***^ | 662.233^***^ | 261.455^**^ | 418.264^***^ |
|  | p = 1.000 |  | p = 0.00000 | p = 0.000 | p = 0.022 | p = 0.007 |
| Tram stop – Tourist site | -26.565 |  | -130.366 |  | -100.967 |  |
|  | p = 0.740 |  | p = 0.107 |  | p = 0.234 |  |
| Rain dummy | 72.005^***^ | 71.938^***^ | 12.281 | 12.333 | 87.719^***^ | 87.800^***^ |
|  | p = 0.007 | p = 0.007 | p = 0.427 | p = 0.425 | p = 0.0003 | p = 0.0003 |
| AM temperature | 27.125^***^ | 27.096^***^ |  |  |  |  |
|  | p = 0.007 | p = 0.007 |  |  |  |  |
| Lunch temperature |  |  | -2.720 | -2.717 |  |  |
|  |  |  | p = 0.546 | p = 0.546 |  |  |
| PM temperature |  |  |  |  | -8.603 | -8.617 |
|  |  |  |  |  | p = 0.209 | p = 0.208 |
|  | | | | | | |
| Observations | 674 | 674 | 674 | 674 | 674 | 674 |
| Log Likelihood | -4,898.226 | -4,907.432 | -4,566.358 | -4,575.495 | -4,838.631 | -4,853.669 |
| Akaike Inf. Crit. | 9,828.452 | 9,834.864 | 9,164.716 | 9,168.989 | 9,709.263 | 9,729.338 |
| Bayesian Inf. Crit. | 9,900.664 | 9,879.997 | 9,236.928 | 9,209.608 | 9,781.474 | 9,778.984 |
|  | | | | | | |
| *Note:* | ^*^p<0.1; ^**^p<0.05; ^***^p<0.01 | | | | | |

**S4 Table.** Results of a “Null hypothesis” test for the model, where results are estimated based on constant built environment and updated built environment data, with sensor dummies (left) and without (right). Prediction accuracy measures for 2015 using a model calibrated on 2014 data are presented.

| **Peak** | **Metric** | **With sensor dummies** | | | **Without sensor dummies** | | |
| --- | --- | --- | --- | --- | --- | --- | --- |
|  |  | **SVR (updated BE)** | **SVR (constant BE)** | **Change (%)** | **SVR (updated BE)** | **SVR (constant BE)** | **Change (%)** |
| AM | R2 | 0.78 | 0.74 | -5.1% | 0.77 | 0.73 | -5.2% |
|  | MAE | 0.27 | 0.22 | -18.5% | 0.33 | 0.37 | 12.1% |
|  | RMSE | 0.48 | 0.53 | 10.4% | 0.57 | 0.62 | 8.8% |
| LUNCH | R2 | 0.84 | 0.82 | -2.4% | 0.78 | 0.76 | -2.6% |
|  | MAE | 0.27 | 0.29 | 7.4% | 0.32 | 0.34 | 6.3% |
|  | RMSE | 0.4 | 0.42 | 5.0% | 0.46 | 0.49 | 6.5% |
| PM | R2 | 0.77 | 0.73 | -5.2% | 0.71 | 0.66 | -7.0% |
|  | MAE | 0.33 | 0.36 | 9.1% | 0.42 | 0.45 | 7.1% |
|  | RMSE | 0.59 | 0.63 | 6.8% | 0.67 | 0.72 | 7.5% |

**S5 Table.** Descriptive statistics of variables used in calibration.

| **Variables (June 2014)** |  |  |  |  |  |  |  |  |  |
| --- | --- | --- | --- | --- | --- | --- | --- | --- | --- |
|  | Mean | Median | Min | Max |  |  |  |  |  |
| **Dependent variables** |  |  |  |  |  |  |  |  |  |
| AM peak (sensor ped. count) n=33 | 889.1 | 607.5 | 107 | 3086 |  |  |  |  |  |
| Lunch peak (sensor ped. count) n=33 | 1157.2 | 810.5 | 177 | 3620 |  |  |  |  |  |
| PM peak (sensor ped. count) n=33 | 1304.8 | 955 | 144 | 3592 |  |  |  |  |  |
|  |  |  |  |  |  |  |  |  |  |
| **Independent variables (estimated flows)** | | |  |  | **Origin weight** | **Destination Weight** | **Beta** | **Radius (m)** | **Detour Ratio** |
| Employment – Train station | 1079.2 | 249.8 | 0.0 | 9721.4 | Jobs & students | Nr of lines and multiplier over tram | 0.001 | 800 | 1.15 |
| Employment – Tram stop | 709.9 | 412.9 | 2.4 | 3370.0 | Jobs & students | Nr of lines | 0.002 | 800 | 1.15 |
| Employment – F&B | 582.2 | 442.4 | 3.6 | 3805.6 | Jobs & students | Seat count | 0.002 | 800 | 1.15 |
| Employment – Park | 303.9 | 0.0 | 0.0 | 2718.4 | Jobs & students | Area (sq meters) | 0.002 | 800 | 1.15 |
| Employment – Parking | 241.1 | 113.5 | 0.0 | 1408.7 | Jobs & students | Parking spaces | 0.002 | 400 | 1.15 |
| Employment – Residential | 396.4 | 324.0 | 3.9 | 2072.2 | Jobs & students | jobs or students | 0.002 | 800 | 1.15 |
| Residential – Tram stop | 60.4 | 47.0 | 0.0 | 192.5 | Residents | Nr of lines | 0.002 | 800 | 1.15 |
| Residential – Park | 21.2 | 0.0 | 0.0 | 171.5 | Residents | Area (sq meters) | 0.002 | 800 | 1.15 |
| Amenities – Amenities | 11.1 | 5.8 | 0.0 | 88.7 | 1 | 1 | 0.002 | 800 | 1.15 |
| Tram stop – Tourist site | 0.3 | 0.1 | 0.0 | 5.5 | Nr of lines | Trip Advisor review count | 0.002 | 800 | 1.15 |
|  |  |  |  |  |  |  |  |  |  |
| **Independent variables (weather)** | |  |  |  |  |  |  |  |  |
| Rainfall (in mm) | 2.1 | 0.8 | 0.0 | 7.0 |  |  |  |  |  |
| Rain (dummy) | 0.6 | 1.0 | 0.0 | 1.0 |  |  |  |  |  |
| AM temperature (Celsius) | 11.4 | 11.1 | 8.7 | 14.0 |  |  |  |  |  |
| Lunch temperature (Celsius) | 10.5 | 10.7 | 5.6 | 13.4 |  |  |  |  |  |
| PM Temperature (Celsius) | 9.9 | 9.7 | 6.1 | 12.9 |  |  |  |  |  |

**S6 Table.** Goodness of fit results for Stochastic Gradient Descent (SGD), Random Forest (RF), Bootstrap Aggregation (BAG), Gradient Boosting (GB), and Gaussian Process (GP) models using 10 types of pedestrian flows combined with weather and day-of-week variables, with sensor-level dummies. Top: calibration results on June 2014 data. Bottom: prediction results on June 2015 data.

| **Calibration on June 2014 data (Mon-Fri)** | | | |  |  |  |
| --- | --- | --- | --- | --- | --- | --- |
| **Peak** | **Metric** | **SGD** | **RF** | **BAG** | **GB** | **GP** |
| AM | R2 | 0.89 | 0.99 | 0.93 | 0.99 | 0.92 |
|  | MAE | 0.23 | 0.04 | 0.13 | 0.07 | 0.13 |
|  | RMSE | 0.38 | 0.12 | 0.3 | 0.11 | 0.31 |
| LUNCH | R2 | 0.9 | 0.99 | 0.97 | 0.97 | 0.96 |
|  | MAE | 0.22 | 0.04 | 0.12 | 0.09 | 0.11 |
|  | RMSE | 0.29 | 0.07 | 0.17 | 0.15 | 0.18 |
| PM | R2 | 0.89 | 0.99 | 0.94 | 0.97 | 0.93 |
|  | MAE | 0.25 | 0.05 | 0.13 | 0.11 | 0.15 |
|  | RMSE | 0.35 | 0.1 | 0.26 | 0.19 | 0.28 |
|  |  |  |  |  |  |  |
| **Prediction on June 2015 data (Mon-Fri)** | | | | |  |  |
| **Peak** | **Metric** | **SGD** | **RF** | **BAG** | **GB** | **GP** |
| AM | R2 | 0.76 | 0.73 | 0.77 | 0.66 | 0.78 |
|  | MAE | 0.32 | 0.29 | 0.33 | 0.35 | 0.34 |
|  | RMSE | 0.58 | 0.61 | 0.57 | 0.69 | 0.57 |
| LUNCH | R2 | 0.73 | 0.78 | 0.8 | 0.8 | 0.81 |
|  | MAE | 0.37 | 0.29 | 0.33 | 0.29 | 0.3 |
|  | RMSE | 0.51 | 0.46 | 0.44 | 0.44 | 0.43 |
| PM | R2 | 0.69 | 0.49 | 0.72 | 0.51 | 0.72 |
|  | MAE | 0.44 | 0.51 | 0.39 | 0.49 | 0.39 |
|  | RMSE | 0.69 | 0.89 | 0.64 | 0.88 | 0.65 |
