# Supplementary material for: We shape our buildings, but do they then shape us? A longitudinal analysis of pedestrian flows and development activity in Melbourne
Source: PLoS One. 2021 Sep 21;16(9):e0257534. doi: 10.1371/journal.pone.0257534 (PMC8454953; doi:10.1371/journal.pone.0257534)
Supplement: Attachment — Submitted filename: REV1_response_to_reviewers.pdf [file pone.0257534.s003.pdf]

Dear Editor,

We greatly appreciate the opportunity to revise our manuscript, "*We shape our buildings, but do they then shape us? A longitudinal analysis of pedestrian flows and development activity in Melbourne.*" The feedback provided by you and the reviewers has been valuable and offered a number of strong ideas for improving the manuscript.

We have revised the paper in several key ways. We have revised our criteria for selecting origin-destination flow variables for the model, and updated all the models and output tables and figures accordingly. We have also provided more detail about the application of the model in the Discussion section, expanded the discussion on limitations, and provided several new figures to provide better context to the findings in Melbourne.

We have also, more generally, revised the text to ensure it is as well-written as possible. We believe the paper has benefited greatly from this round of review. Please find below our point-by-point responses to the reviewers' comments. Thank you.

We note that you have indicated that data from this study are available upon request. PLOS only allows data to be available upon request if there are legal or ethical restrictions on sharing data publicly.

> We can share the input data for the model as part of this resubmission, as we already did with one of the reviewers previously.

We note that Figures 1 and 3 in your submission contain map images which may be copyrighted. All PLOS content is published under the Creative Commons Attribution License (CC BY 4.0), which means that the manuscript, images, and Supporting Information files will be freely available online, and any third party is permitted to access, download, copy, distribute, and use these materials in any way, even commercially, with proper attribution.

> We can confirm that all the maps and figures in the paper are original. The basemap layers used in these images are all OpenStreetMap based, which allow CC BY 4.0 use.

Reviewer #1:

The article presents results of a statistical analysis of sensor data for counting the volume of pedestrian flow in Melbourne. The volume of pedestrian flows is correlated with factors of urban life (land use, …) to calibrate the model and test its predictive ability. The importance of trips by

foot is growing and such data are rare but important for planning cities and supporting foot travel. Therefore, in principle, the reviewer supports the publication. However, the presentation of the results is not optimal and the statistical analysis and interpretation of the results also need improvement. The reviewer suggests a revision of the article along the following comments before publication.

1. The central criticism concerns the 11 factors (‘estimated pedestrian flow types: emp_trn, emp_trm, …) considered in the model for the pedestrian volumes. Figures 4 and 5 show that most of the factors seem to have little contribution, or not much importance. In the same time, it is obvious that the factors that have been taken into account cannot be complete. As the authors state in line 173 -176 many factors are not included. The reviewer is aware that here completeness cannot be achieved. However, it would be possible in the statistical analysis to consider that unknown factors (random effects) were neglected and how strong their influence is. It seems that random effects were considered by the authors, see line 196, but it is not explained in detail how these random effects influence the results and how important they are. Furthermore it is surprising that many of the factors (flow types) hardly make an explanatory contribution to the model. I think it would be appropriate to check whether the high complexity (high number of fixed variables) is justified or necessary (e.g. by using the Akaike Information Criterion).

Thank you for evoking the important question about variable inclusion and model structure. Our selection of trip types in the model was guided by the following steps. We first identified a longer, potential list of origin-destination trip types that included 36 pairs (Figure 2). We then analyzed trip-generation data for various different destination types published by the Institute of Transportation Engineers in the US (ITE 2017), which suggested that certain trip types (e.g., to offices, to amenities, to parks, to schools, etc.) may be dominant in the typical US context in the morning and evening peak periods that the ITE data describes. However, the ITE data has no trip generation data related to transit stations available, which we thought would be important in Melbourne. Despite some of the peak hour trips likely being universal in highly developed Anglophone cities in moderate climates (e.g., to offices, to amenities, to parks, to schools), we also questioned the validity of transposing American trip generation rates to the urban context of Melbourne. Therefore, we additionally spoke to several planners and urban designers in Melbourne, AU and discussed which of these trip types appeared most to intuitively describe foot-traffic during the AM, lunch, and PM peak periods to them. These conversations helped us finalize the 10 types of O-D flows that were eventually included in the model (pp. 8-9).

We have slightly revised our variable inclusion criteria as follows. Each of the 10 flows was first tested alone for correlation with sensor counts. We have added a Supp. Table 6 to describe these. Then, for inclusion in the models, we only included these flows that showed a 5% or greater bivariate (positive) correlation with observer counts during a given period

(AM, Lunch, or PM) in a given calibration year. The list of variables in the prediction model now therefore shifts somewhat from year to year (see box-plots in Figure 5).

Figure 5 box-plots illustrate the feature importance of each variable that is included in the mode. The feature importance is the appropriate statistic to measure the impact of each individual variable on the model prediction accuracy.

For the comparative multi-level model (MLM), which is reported in Table 3 and Supp. Table 8, we produced a more parsimonious setup, where some of the flows were further eliminated due to multicollinearity that led to flipping signs (which do not have any theoretical or conceptual basis). However, this multi-level model is presented only for comparison here and our main machine learning SVR model was chosen because it addresses multicollinearity and potential nonlinearity directly. We have further clarified these points on pp. 14.

Regarding random effects, both our MLM and Machine Learning models were tested both with and without random effects for sensors (as mentioned on pp. 14, 16). The MLM model outputs are also shown in Supp. Table 8. We present in Table 4 the specific impact that sensor level dummies can have on the model and highlight in the paper that in order to predict foot-traffic on streets other than those with sensors, we need to use a model without sensor level dummies. We have therefore presented all the following predictions (Figs 4, 5, 6, 7 and Table 5) using a model where sensor-level dummies are omitted.

2. More details should be provided in the presentation and discussion of the CLUE data used for the model calibration. In particular, information about the spatial resolution of the data would be important to ensure the reproducibility of the study and to be able to evaluate the results.

We have added additional information about the CLUE data on pp. 10 and included a reference to the CLUE website that provides further detail. We have also added Table 1, which illustrates year-on-year changes observed in the CLUE data.

3. Line 33 – 38: On the one side the authors conclude that an update should take place every two years. One the other hand they found that structural changes influence pedestrian traffic. This seems strange. What if in two years no structural changes occur. Or if many structural changes happen in the period of one year?

We note that calibrated coefficients can change over time due to a number of conditions that remain unobserved in our model--e.g., demographic changes in the city, annual shifts in tourism activities, cultural changes in walking activity that may result from policies that promote more walking, etc (pp. 25).

We have clarified and revised our stance on recalibration on pp. 23-24, where we show that after the first year of predictions, model accuracy declines by ~12% and stays at that level for the following 3 years. We therefore suggest it would be best to recalibrate coefficients annually if the model were used in an official context. We also note that, in principle, re-calibration could be continuous and automated based on updated pedestrian counter data, but, in practice, an annual calibration seems to make most sense due to CLUE data, which is currently updated annually. Also, from a policy standpoint, if this type of a model were implemented as an official "pedestrian impact assessment" tool, it seems more desirable to have stable coefficients for each year consecutively. Continuously changing coefficients could cause unnecessary confusion in planning practice.

As to the Reviewer's point on 'no structural change', there is no practical scenario where no new building activity occurs in a year, let alone 2 or 3 years. Cities like Melbourne change constantly. However, theoretically, if the building data (CLUE) showed no change at all in a year, our predicted pedestrian flows would also remain almost constant--estimates would only be affected by weather, day and hour variables, whose effects are relatively small. We have added an additional table to Supplementary Materials (Supp. Table 9) in which we compare how the prediction fit changes when new built environment changes are included (referred to as updated BE) and not included (referred to as constant BE). The inclusion of BE variables consistently produces a more accurate prediction.

4. Line 71 and other places in the manuscript. 'Longitudinal' is a term referring to spatial information. To my understanding the authors use it to describe a property in time. Is this intended?

Both the CLUE data and the pedestrian count data are collected over time. The term "longitudinal" is used in the paper to refer to temporal changes in pedestrian flows and the built environment.

5. Line 104 to 106: This filtering in the data seems important to me and I expect the results to be very sensitive. It should be investigated whether the chosen filtering is justified and how the results change when the filtering is changed.

We assume that the Reviewer is referring to our adjustment to the Betweenness index here. In predicting trip distribution in spatial networks, only a small fraction of nodes produce meaningful interactions. We have cited the National Household Travel Survey and Institute of Transport Engineers (ITE) trip generation studies on pp. 8 to illustrate what the most common trip types are during different times of the day, which we used to select candidates for inclusion in our model. The method through which we ultimately selected O-D pairs to be included in the model is explained on pp. 9.

6. Line 114:. What is meant with elastic? Larger variability? Of what quantity? More precision in the language is necessary.

In addition to using the Huff probability model to allocate trips to competing destinations k, we include a Gravity Accessibility term, which ensures that the number of trips from [j] to [k] also depend on the wight and proximity of destination k:

$$Trips[j,k] = W[j] \cdot Probability[j,k] \cdot \frac{W[k]^{\alpha}}{e^{\beta \cdot d[j,k]}}$$

Consider for example a single trip origin (building) with 10 people in it, and a single destination within its walking radius, such as a restaurant with a size of 1,000 sqft. Depending on how far and big the destination is, we find the rate of trips sent to this destination. Now, if another business establishment is added to the destination building (adding a weight of 1,000 sqft), then that building obtains a new, larger weight (2,000sqft). This would result in allocating more trips from the same origin, even when origin weight stays the same at 10 people. Our elasticity effect ensures that when destination weights expand, or when they are closer to the origin, trip generation increases.

7. Line 121: '… idiosyncratic …' What is meant by the term? Can this be specified. E.g. Individual criteria of route choice, such as safety, comfort, …

We have added more detailed explanation on p. 6.

8. Line 235 to 237: unprecise terminology: '… multiple forces …' behavior or group dynamics is not a force.

We have clarified the language on p. 17.

9. Line 300: The study distinguishes between trips from home to work and trips from home to access public transit. But a trip from home to work can also include public transit. It is not clear to me how the model accounts for this duality.

We have included the following explanation to this on p. 21: "Even though walking trips from homes to transit stations, transit stations to employment locations and directly from homes to employments locations may illustrate different legs of trips headed to work in the morning peak period, we treat them separately here because our model does not seek to

estimate individual agents' daily activities as complete trips, but rather pedestrian flows on individual street segments."

10. Figure 4: The abbreviations used in the y-axis should be defined clearly

Thanks for pointing this out, we have updated all figures and abbreviations accordingly.

11. Table 3: What is the reason that the predictive power of the models change for AM and PM but less for the Lunchtime Periode?

Thanks, this is an interesting observation, which we also note when commenting on Table 2 on pp. 12. We now also draw attention to this in pp. 23.

Reviewer #2:

Overall, the manuscript (titled: We shape our buildings, but do they then shape us? A longitudinal analysis of pedestrian flows and development activity in Melbourne) aims to make some contributions to forecasting of the effect of urban form, land uses, amenities and pedestrian walkway layout as well as climatic conditions changes over time using pedestrian flows. Three different time period are investigated based on updated fine grained built environment data available in Central Melbourne. The topic is appropriate for the journal PLOS ONE and will interest urban planner, urban designer, geographers, transportation planners, and policy makers. The manuscript is well-written; however, some issues remain to be addressed before the paper will be ready for publication.

It is known that pedestrian flows over time/space are well correlated. It would be good to have a sense of these correlations over time/space and to understand how the model does or not better than the null hypothesis i.e. pedestrian flows are staying the same/have very high correlation between the calibration year and the predicted year.

Thanks for this observation. We have added an additional table to Supplementary Materials (Supp. Table 9) in which we compare how the prediction fit changes when new built environment changes are included (referred to as updated BE) and not included (referred to as constant BE). The inclusion of BE variables consistently produces a more accurate prediction. However, as expected, the change is not substantial since the built environment only changes marginally year to year. We have also added a new Table 1 that illustrates how much BE change is observed in each year in our study area.

The paper does not make clear what/where is changing in the built environment how much is changing whether land use changes are marginal or not, how this affect or not pedestrian flow distribution or whether it is for example the pedestrian layout change or not and where during the calibration year and the predicted year and over the overall available period to have a sense of the magnitude of change.

> Thanks for emphasizing this. As noted in the previous comment, we have added Table 1, which summarizes the extent of built environment change each year. Additionally, we have added a new Figure 5, which illustrates BE change on a map between 2014-15 and demonstrates how the pedestrian flows are predicted to change due to the observed BE changes.

With such detailed data set from the same reliable source, it would be good to know how the year-on-year means is changing or not and whether it is fully attributable to BE change or to exogenous factors. This is picked up in the last part of the paper – but not shown anywhere to begin with.

> Thanks for this suggestion. We have added a Table 2 to the paper, which shows the yearly sensor-level summary statistics, and some discussion on this in pp.12.

Please give the BEs and list of independent variables and their descriptive statistics. Whenever available the coefficients should also be given. Is this a ML black box model? The paper mention various weighting can these be described systematically.

> As noted above, the descriptive statistics of the built environment variables are now shown in Table 1. We have also added the ML model coefficients to Supp. Table 8.
>
> The weighting refers to the numeric attributes of trip origin and destination points used in the modified betweenness algorithm (Equation 1), which are listed for each origin/destination type as part of Figure 2. For instance, parks are weighted by their land area--instead of treating each park as if they were identical, we assume larger parks to constitute a larger attraction for pedestrians. Similarly, trips originating at residential buildings are weighted by the number of residential units in each building etc.

Give indication of the pedestrian network area (cropped on figure 3) and of UNA running times.

> We have added Figure 3 as a location map to the paper. The UNA running times range from a couple of hours to seconds, depending on the number of origins and destinations and the detour ratio used. The most taxing analyses were residents to jobs, amenities to

amenities, and jobs to amenities, each of which had over 7,000 origins and destinations; these took a couple of hours on a standard desktop computer to estimate.

Measuring overall model success with RMSE implies that scale information is preserved. RMSE does not necessarily increase with the variance of the errors. RMSE increases with the variance of the frequency distribution of error magnitudes. Does the model claim to measure overall demand? i.e. indirect linkage caused by external economic on both the overall levels of flow and the BE changes themselves? Dependencies might occur only directly. What is predicted needs some clarification early on.

Our models do not measure overall demand, since we do not have information on external factors (e.g., macro-economics). Since we are interested in understanding the micro-level changes in pedestrian flows (e.g., at the sensor-level or the segment-level) due to changes in the built environment, major changes in macro-economic conditions from year-to-year will have to be captured by random effects to the extent possible. Should a substantial shock occur to the system, our model estimates will suffer from some error, but that would be true for any other similar modeling framework. More complex and sophisticated land use and transportation interaction (LUTI) models may be better suited to capturing effects of external changes (e.g.computable general equilibrium models), but they have their own shortcomings (and a whole other literature).

The comparison of the BE actual change versus the share of each source in the model would be of interest. This is key in a forecast model.

We have added Figure 6 to the article, which illustrates the relationship between built environment changes and corresponding changes in foot-traffic predictions. We have also added language throughout the article to comment on this relationship. For instance, on pp. 26 we refer back to Table 1 to illustrate how the relative decrease in amenity-to-amenity trips during the lunch period is in part explained by the relative decrease in amenities per capita over the years.

The model claims to combine the Huff model with Betweenness, could the authors give the formula used. This combination could give some rather non-standard elasticity effects unless done right.

The Betweenness index is estimated as:

$$Betweenness[i]^{r,dr} = \sum_{j,k \in G-\{i\}, d[j,k] \leq r \cdot dr} \frac{n_{j,k}[i]}{n_{j,k}} \cdot W[j] \cdot \frac{W[k]^{\alpha}}{e^{\beta \cdot d[j,k]}}$$

, where Betweenness $[i]^{r,dr}$ describes how many "plausible" walks from surrounding origins [*j*] to destinations [*k*] in a network *G* pass through location [*i*], such that the maximum distance between [*j*] and [*k*] is less or equal to search radius *r* multiplied by the given detour ratio *dr*. If weights W are attached to origin points [*j*], then the number of walks routed from [*j*] to [*k*] corresponds to W. W[k] shows destination attractiveness with alpha as coefficient.

Betweenness is integrated with the Huff model and a destination elasticity term as follows.The Gravity accessibility between a particular origin [j] and destination [k] within a network radius "r" is given as:

$$Gravity[j,k]^{r} = \frac{W[k]^{\alpha}}{e^{\beta \cdot d[j,k]}}$$

, where W[k] is the weight or attractiveness of destination [k] and α is a variable exponent that can control the effect of destination attractiveness on the outcome; d[j,k] is the network distance between origin [j] and destination [k] and β is the exponent that defines the steepness of the distance decay function. This represents a typical gravity accessibility index (Hansen 1959; Handy and Niemeier 1997).

The sum of accessibilities from origin point [j] to all available destinations [k] that are available within the specified network radius "r" around it, is given as:

$$Gravity[j]^{r} = \sum_{j \in G-\{j\}, d[j,k] \leq r}^{n} \frac{W[k]^{\alpha}}{e^{\beta \cdot d[j,k]}}$$

The probability that a person at origin point [j] will patronize a particular destination point [k] is given as a ratio between [j]'s gravity accessibility to that particular destination divided by [j]'s sum of gravity accessibilities to all available destinations, including [k]:

$$Probability[j,k] = \frac{Gravity[j,k]}{Gravity[j]^{r}}$$

Since the gravity calculations are determined by both the proximity of the destinations and the attractiveness of the destinations (e.g. destination weight), the probability of visiting any destination is determined by both factors simultaneously as well as the presence of competing facilities. This probability is constrained between 0 and 1.

Given these definitions, the number of trips that are sent from origin [j] to a particular destination [k] is found by multiplying the weight of [j] with the probability [j,k]:

$$Trips[j,k] = W[j] \cdot Probability[j,k]$$

In order to add a demand elasticity to supply effect (having walking access to more destinations increases the probability of trips to each of the destinations), the number of trips from $[j,k]$ should also depend on j's accessibility to k type of destinations:

$$Trips[j,k] = W[j] \cdot Probability[j,k] \cdot \frac{W[k]^{\alpha}}{e^{\beta \cdot d[j,k]}}$$

The accessibility term appears twice in the equation—once to determine the probability and then to control trip elasticity at the end of the equation. This is necessary since Huff's original probability formulation is agnostic to how far or accessible destinations are—all origin weights will always be distributed among available destinations. If we compare two households, each with access to three bus stops, but one's bus stops are at the edge of the search radius, while the others are right next to the house, Huff's probability will assign about 1/3 probability to each stop in either case (depending on relative accessibility of each). The additional term added here ensures that trip volumes are elastic with respect to destination accessibility. Further away and less attractive destinations are allocated fewer trips. The elasticity term ensures that increasing destination weights can also increase trip volume, even if origin weights are kept fixed.

In the city centre of Melbourne, it is surprising to see no relationship between non-food and food business in Figure 2, could this choice be explained/justified?

We have included amenity-to-amenity trips in the model, but not separately amenity-to-F&B simply because the two are highly correlated with each other. Retail and service shops are typically intertwined with F&B establishments on streets. However, we do note the relevance of employment-to-F&B walks in the paper for both lunch and PM results (Figures 6, 7).

In the study area, there are well used publicly accessible indoor multi-level pedestrian paths which are well connected with the outdoor network, could the authors clarify what is the extent of that network and in what way these omitted parts of the pedestrian network and flows affect the proposed model/results?

Our model features outdoor pathways and cross-walks only; we have added a note about this on p. 11. Indoor factors are taken into account by assigning appropriate weights to buildings where trips start or end. Adding indoor links to the network, which indeed is uniquely available in Melbourne would likely increase the resolution of the analysis, but not alter the overall results. We chose to keep the model outdoors-only for two main reasons: first, because there are no pedestrian count sensors available indoors as far as we know, and second, to make the model more readily replicable in other cities (most cities are unlikely to have indoor networks documented). In principle, the model could be readily extended to include indoor networks in future research.

It is unclear why the model should be recalibrated every two years. Hopefully, the information requested above will also help articulate why it is so.

We have added more clarifications about recalibrating the model onto pp. 23. Briefly, recalibration over a certain time period is generally needed because behavior coefficients in the model can change over time due to a number of conditions that remain unobserved in our model—e.g., demographic changes in the city, annual shifts in tourism, cultural changes in walking activity that may result from policies that promote more walking, etc. In theory, if the CLUE data are to be updated continuously (e.g., every day or week), just like the pedestrian counter data already is, it would be possible to recalibrate the model continuously. But, in practice, this is unnecessary and probably undesirable. If such a model were to be taken as an "official" framework for conducting pedestrian impact assessments of individual development projects, it would be necessary to have a stable model throughout at least a year and a clear understanding when new model coefficients are annual released for all to use (e.g., January 1st). We have updated the paper to suggest that the model be calibrated annually.

Figures 4 and 5 are unreadable (low resolution) and the variables are not defined/listed anywhere.

This appears to be an issue of the PLOS ONE online system reformatting the figures to lower resolution. The original figures we upload are all higher resolution. We have received similar comments for other submissions to PLOS ONE as well. Could PLOS ONE provide the reviewer 'raw' uploaded figures, if possible?

Please justify the specific radii size (800m). We don't think that the US Transport Board reference is adequate when effectively the model claims for a context sensitive approach (Melbourne's travel surveys?).

We have discussed the appropriateness of cutoff radii for walking with Melbourne planners and think 800m is indeed appropriate for Melbourne and other highly developed cities in similar climates. We have also added a citation to Eady and Burtt (2019) on pp. 8, who found that in Melbourne's inner suburbs, median pedestrian trip length was 563m and the average 770m. We expect CBD trips to be shorter.

Please note that the model includes an important distance decay rate, which assumes that the likelihood to walk drops with distance. While 800m represents the cutoff radius beyond which we no longer consider trips (surely some exist, but they tend to be a minority and are unlikely to affect overall foot-traffic volumes significantly), shorter trips are assumed to be far more likely than longer trips.

Context sensitivity in the model primarily comes from calibrated flow coefficients, which likely vary in every city. Here we have calibrated the model specifically on Melbourne's pedestrian counts.

There is still missing reference to layout change and its association with flow changes over time in this paper and software that provide the same.

It is not clear to us what Reviewer #2 means by "layout change" here. If this comment is addressing how the spatial layout of the city can affect the model predictions, then this is indeed a central factor in the model--as the location or weights of origins, destinations or walk networks between them change, model predictions also change. Newly added Tables 1 and 2, illustrate the extent of built environment change and pedestrian activity change observed each year. Newly added Figure 5 illustrates how changes in 2014-15 development predict changes in pedestrian activity.

What do the results tell us about the broader implications of pedestrian flow prediction?

We have added more discussion about potential use cases and policy implementation to the Discussion section on pp. 27-28.

A couple of points regarding ergonomics of the model – within a general design process of street improvement and change or monitoring where does the model stand? Is it more likely to be used for exploring multiples options during early design exploration stage, or is it on a par with micro-simulation model?

In short, elaborate why the model or the tool is useful, and how the model (or tool) can be effectively used in the urban/transport planning and design practices, for example to appraise

layout change (e.g. before and after Stanton St. or Bourke St. redevelopment, management change for example conversion to walking street (7 between 2014-17 – Melbourne walking plan) vs land use or amenities changes.

> The additions in the discussion section on pp. 27-28 hopefully address this question. We think an ideal use case for the model would involve a city adopting the methodology to publish an "official" foot-traffic estimate for all streets within a given year (much like Annual Average Daily Traffic or AADT metrics are available for all street segments in numerous cities). This would require a single calibration each year, with the segment-by-segment results published freely online. We additionally mention that a more elaborate, interactive web-mapping platform could contain the functionality to overwrite plot characteristics (land use and density) to explore how such changes could impact pedestrian flows around the location. Though the results of such scenario tests could take some time (e.g., minutes, possibly hours), the back-end results could be returned to the users as map layers with data tables when complete.

In the current state of the paper, it remains difficult to interpret results and value them, especially deviations to counts and the absence of level of service. The paper would increase its relevance when comparing results with another model (beside null model). For comparison, in theory there are multi-modal models (and therefore pedestrian models) available, including flow forecast, would it be possible to add a variation / modification of provided model, with corresponding results. Given the data set required it remains unclear if all components and considered methods are necessary as a whole for pedestrian forecast and thus if the model is really transferable as most cities in the world do not have such comprehensive data set.

> While a comparative analysis with other multi-modal transportation models would be interesting, this remains beyond the scope of this study and part of future research. We do think the model can be transferred to other city contexts relatively readily. The land use data -- residential distribution, job locations, amenities, and transportation networks are generally available and openly accessible data in the North American context as well as a number of EU countries. What is usually lacking are accurate pedestrian count data that Melbourne has. However, we note in the paper that even manual pedestrian counting surveys could be used to collect calibration data, though automated counts would of course be preferable due to their longitudinal nature. We have added two additional references to automated pedestrian and bike counting reviews in the last paragraph of the article on pp. 30
