# Supplementary material for: We shape our buildings, but do they then shape us? A longitudinal analysis of pedestrian flows and development activity in Melbourne
Source: PLoS One. 2021 Sep 21;16(9):e0257534. doi: 10.1371/journal.pone.0257534 (PMC8454953; doi:10.1371/journal.pone.0257534)
Supplement: Attachment — Submitted filename: Response_to_Reviewers_REV2.docx [file pone.0257534.s004.docx]

**­­Response to Reviewers (PONE-D-20-36700R1)**

*We would like to thank the two reviewers for their constructive feedback. We have revised the manuscript in an effort to address your comments and incorporate your suggestions. Our responses to your specific comments can be found below. We hope that the revised draft will make for a stronger submission.*

**Reviewer #1**

The comments were essentially taken into account by the authors. Here are just two final feedbacks on the responses to the reviewer's comments:

4. Line 71 and other places in the manuscript. ‘Longitudinal’ is a term referring to spatial information. To my understanding the authors use it to describe a property in time. Is this intended?

Answ: Both the CLUE data and the pedestrian count data are collected over time. The term
“longitudinal” is used in the paper to refer to temporal changes in pedestrian flows and the
built environment.

Repl to the Answ: This is no answer to my point. ‘Longitudinal’ is a term referring to spatial information (NOT temporal) and thus not used properly in the manuscript.

***Authors’ Response:*** *Thank you. We believe this is a misunderstanding. Longitudinal data, sometimes also referred to as panel data, track the same sample over different points in time. Our CLUE data and pedestrian count data are longitudinal (they vary temporally over time) and it is their temporal variation that we are referring to in the paper. The term ‘longitudinal’ is commonly used in transportation, econometric and planning literature in this context.*

Line 114: What is meant with elasticity? Larger variability? Of what quantity? More precision in the language is necessary.

Answ: In addition to using the Huff probability model to allocate trips to competing destinations
k, we include a Gravity Accessibility term, which ensures that the number of trips from [j]
to [k] also depend on the wight and proximity of destination k:

[, ] = [] ∙ [, ] ∙ [] ∙ [,]

Consider for example a single trip origin (building) with 10 people in it, and a single
destination within its walking radius, such as a restaurant with a size of 1,000 sqft.
Depending on how far and big the destination is, we find the rate of trips sent to this
destination. Now, if another business establishment is added to the destination building
(adding a weight of 1,000 sqft), then that building obtains a new, larger weight (2,000sqft).
This would result in allocating more trips from the same origin, even when origin weight
stays the same at 10 people. Our elasticity effect ensures that when destination weights
expand, or when they are closer to the origin, trip generation increases.

Repl to the Answ: Many thanks for the explanation how the term ‘elastic’ should be understood. Please reconsider whether this explanation is of interest for potential readers, too! Or better choose a more appropriate term. Would ‘variabality’ of the model wrong?

***Authors’ Response:*** *Following your suggestion, we have added this explanation to the paper in Supplementary Note 1 and a reference to it on page 6. The term ‘elasticity’ is frequently used in transportation models to describe how a modeled trip generation responds to changes in destination availability. We have therefore preferred to use the term for consistency.*

**Reviewer #2**

Thank you for addressing most of the comments and criticisms. Three major criticisms remain. Two are about the selection of the factors considered in the model:

1. the omission of the role of the pedestrian outdoor pathway layout in pedestrian route choice assignment: Shatu, et al. (2019) in a small study in Brisbane, Australia (N= 178) have shown that pedestrian route choice is mostly an angular distance minimization and secondarily Euclidean minimization. The pedestrian model proposed in Melbourne should at least include a route choice assignment that is aligned with this recent study of pedestrian route choice in Brisbane. Moreover, a large-scale study (Bongiorno, et al., 2021) using phone traces in the US (Boston and San Francisco, N= 552,478) also shows that pedestrian route choice is “vector-based” i.e., mainly angular “a cost that depends on the angular deviation of the street segment from the destination” and that there is asymmetry when OD are swapped. The paper shows that including this angular approach to pedestrian route choice increase model predictive power by 35%. The route choice assignment following shortest angular path that would include 15% detour would be very different than shortest Euclidean path that include 15% detour used in the proposed model. Given that UNA the software used in the paper includes directness metrics we expect that this pedestrian route choice preference and pedestrian outdoor pathway layout factor be included in the model and compared with the current route choice assignment based on Euclidean distance.

***Authors’ Response:*** *Thank you for these suggestions and references to the “turns” versus “distance” debate in pedestrian navigation literature.*

*In transportation literature, distance or travel time are commonly found to be the primary transportation cost attributes, with turns along the route, as well as other route attributes (e.g., businesses passed, vegetation, etc.) playing an important role as well. We have reviewed both of the recent studies suggested and included them as references within the paper. Additionally, we have also used the same dataset as Bongiorno et al. (2021) to estimate the relative impact of route length and turns on route choice in both San Francisco and Boston and illustrate the findings below (we happen to have access to the same datasets they used).*

*First, as expected, distance and turns are collinear with each other (r=0.43 in San Francisco and r=0.59 in Boston). When tested alone, we find distance to be a slightly stronger predictor of route choice in both Boston and San Francisco, using a path size logit model (Ben-Akiva and Bierlaire, 1999), as shown in Table 1 below. We should note that the methodology for analyzing the importance of length and turns here is based on random utility theory and differs from the much simpler correlation analysis presented in the two papers above.*

*While it is clear that both distance and turns play an important role in pedestrian route choice, we believe our approach of using distance and distance-based detours for route generation is justified and consistent with prior pedestrian mobility research. That being said, we do agree that future research should try to implement algorithms that account for both distance and turns concurrently (perhaps using weights that will have to be empirically tested) for route generation. At present, the UNA tools we used for route generation do not have the capability to generate routes based on both distance and turns and such implementation for this article would involve a major effort and completely re-doing the analysis, which we believe is neither justified nor necessary.*

*We have included further comments about these questions (in Materials and Methods on page 6 and in the Discussion on pages 29-30) of the article. We suggest that a lengthier treatment of the topic should be addressed in a separate, future research article.*

*Table 1. Path size logit models for pedestrian path choice in San Francisco (top) and Boston (bottom).*

Ben-Akiva, M., & Bierlaire, M. (1999). Discrete choice methods and their applications to short term travel decisions. In *Handbook of transportation science.* (pp. 5–33). Retrieved from <https://link.springer.com/chapter/10.1007/978-1-4615-5203-1_2>

2. The pedestrian flow model proposed is longitudinal, yet it seems that the pedestrian outdoor pathway network remains the same from the base model and for the forecasted years. Could the authors provide information about the pedestrian outdoor pathway network layout between the baseline and the successive forecasted years: as to whether pedestrian outdoor pathway network layout changed and by how much/where? Whether if pedestrian outdoor pathway network layout changed the modelling assumed that it was the same or not? What are the possible consequence on the modelling?

*We used the pedestrian network published by the Melbourne City Government (*[*https://data.melbourne.vic.gov.au/Transport/Pedestrian-Network/4id4-tydi*](https://data.melbourne.vic.gov.au/Transport/Pedestrian-Network/4id4-tydi)*) timestamped 2018 as the baseline for all of our models. Since the pedestrian network we used was outdoor only (including sidewalks, street crossings, etc.), changes in the network structure itself over time are likely minimal during our study period. Indeed, new projects and developments that occurred between 2014 and 2019 very likely added private but publicly accessible indoor environments (such as courtyards, etc.) but the layout of streets and sidewalks is less affected by such shifts. We have added a note to the discussion section about this (page. 30) and suggested that future research could further examine how the network itself changes over time.*

3. Thank you for providing and extensive specification of the betweenness index and the definition of elasticity which is to say the least is unorthodox: e.g., is it possible that with an alpha = 1 then increasing a destination weight can potentially deprive other destinations of trips, which shouldn’t happen in a fully elastic model. It seems that the betweenness index is the same that was used in Sevtsuk A., 2021, JAPA. It was then stated that “the model does not presently incorporate destination count elasticity, which should be addressed in future work.” Is this issue addressed in this paper? It should. If not, the same disclaimer should be included, and the originality contribution of this paper would be somehow limited to the longitudinal modelling in Melbourne.

*Yes, this paper implements the same underlying pedestrian routing model as Sevtsuk (2021) used in JAPA (as stated on page 4) and does not claim any changes to the pedestrian routing model specification itself. The key contributions of this paper lie in (a) using a much more precise, longitudinal dataset of pedestrian counts from automated sensors as dependent variables, (b) developing predictive models where the accuracy of the predictions can be validated using observed data in the following years, and (c) using more innovative and consistent machine learning estimation techniques to tackle issues of multi-collinearity and non-linear relationships in the data. We have further clarified this in the paper on pages 5, 6.*
