# Supplementary material for: We shape our buildings, but do they then shape us? A longitudinal analysis of pedestrian flows and development activity in Melbourne
Source: PLoS One. 2021 Sep 21;16(9):e0257534. doi: 10.1371/journal.pone.0257534 (PMC8454953; doi:10.1371/journal.pone.0257534)
Supplement: Attachment — Submitted filename: Response_to_Review_REV3.docx [file pone.0257534.s005.docx]

**­­Response to Editor (PONE-D-20-36700R2)**

*We are delighted that the two reviewers are satisfied with the revised paper and would like to thank them for their constructive feedback. Please see our responses to your additional editorial questions and comments below.*

**Editor**

Please review your reference list to ensure that it is complete and correct. If you have cited papers that have been retracted, please include the rationale for doing so in the manuscript text, or remove these references and replace them with relevant current references. Any changes to the reference list should be mentioned in the rebuttal letter that accompanies your revised manuscript. If you need to cite a retracted article, indicate the article’s retracted status in the References list and also include a citation and full reference for the retraction notice.

*We have reviewed the reference list and corrected a few URL links that did not seem to work due to broken DOI links earlier. We have made sure all links are now active and correct. We also found that a couple of paper had been referenced twice and corrected that.*

Because two reviewers provided positive comments, I provide a suggestion of minor revision. But I am confused by several issues when I read the paper. Please carefully address the following issues. If the revision is still unsatisfactory, I might need to find additional reviewers for another round of review.

First, in the "statistical methods" section, what are your dependent variables? & independent variables? How are they defined and measured? Are they street-link based, or sensor-based? How many samples in your models? Also, a descriptive statistics table should be provided. It is hard to judge the soundness based on your current description on methods, let alone to replicate your methods.

*A description of the variables was included in the ‘Materials and Methods’ and ‘Data’ section previously. For clarity, we have now called out a subsection entitled ‘Modeling Pedestrian Flows’ and moved the calibration model description from the ‘Data’ section to the ‘Statistical Methods for Model Calibration’ section. Independent variables, which predominantly comprise estimated pedestrian flows, are described in the ‘Modeling Pedestrian Flows’ section. Model parameters are also described in ‘Modeling Pedestrian Flows’ section, and an additional table with descriptive statistics for independent variables has been added as Supp. Table 10.*

*As discussed in the ‘Statistical Methods for Model Calibration’ section, observed pedestrian counts from Melbourne’s automated counters are used as dependent variables. Independent variables include predicted pedestrian flows at each sensor location between up to ten types of origin-destination pairs, as well as additional variables for weather and time of day. This has been described in the paper since the original submission. We had also provided the origin and destination weights in Figure 3 and noted this in the figure caption for replicability. We have now additionally included these weights and corresponding detour ratios used in the pedestrian flow model in Supp. Table 10.*

*We have added an emphasis in ‘Modeling Pedestrian Flows’ (p. 12), that “Appropriate pedestrian flow types, their corresponding O-D pairs and assumed distances and detours that capture most of the observed variation in pedestrian flow can vary in different contexts and land-use patterns”. The model should thus not be replicated with the same set of O-D flows and specifications in different urban contexts—these need to be considered relative to each study context. The elements of this study that are directly replicable are the model framework, and its calibration and validation methods.*

*The criteria we used for O-D flow inclusion in each of the three travel periods (AM, lunch, PM) are described in the ‘Statistical Methods for Model Calibration’ section on p. 13.*

Second, as the built environment is a key word of your study, you need to provide a theoretical debate on why and how selecting such built environment variables, and what are their associations with walking behaviors? In this case, a literature review is needed to add in the Introduction or variable section. There are lots of related studies; you may refer to Handy, 2018, Enough with the “ D ’ s ” already—Let ’ s get back to “ A .” and Zhang et al., 2020, Nonlinear effect of accessibility on car ownership in Beijing: Pedestrian-scale neighborhood planning.

*We have referred to literature on pedestrian route choice variables in the Introduction (p.3-4), in ‘Modeling Pedestrian Flows’ (p. 9 and p. 10) and on p. 17. We have now supplemented additional literature references to the ‘Introduction’ section (p. 4), which mentions some of the prior “aggregate” planning studies on walkability using the 3Ds and other aggregate neighborhood-level variables. We have also incorporated a reference to the magazine article by Susan Handy you recommended as the kind of approach she calls for aligns well with the model we implement in this article.*

*Regarding non-linearity, we have added two references to the potentiality of non-linear functional relationships between walking activity and built environments form prior studies, including Tao et al. 2021 and Zhang et al. 2020 (p. 15). We also added Supp. Figure 2, which illustrates non-linearity in the partial dependence plots of some of our variables with respect to the outcome in the final SVR model we chose.*

*Given the already long list of references in the paper (we currently stand at n = 71), we feel that a separate literature review section is not needed. Instead, various existing approaches are referred to throughout the ‘Introduction’ and ‘Methods and Materials’ sub-sections.*

Third, the modeling process reported in Pages 17-26 are difficult to follow. I would suggest creating a table to show your modeling settings one by one in advance (e.g., in the method section), instead of reporting your modeling processes in the result section.

*There are no modeling settings per se, except for the weights and detour ratios we used to estimate pedestrian flows and which are reported in Supp. Table 10. The Statistical Methods section explains our choices with specifying and selecting Machine Learning models.*

*We think that consolidating the discussion pertaining to pedestrian flow variables in the ‘Modeling Pedestrian Flows’ section, and the calibration and predictions model details in the Statistical Methods section helps clarify the setup for the reader.*

*We also added the following to address your question about the standard for model selection to p.15 : “In order to address overfitting concerns related to machine learning models, we employed 10-fold cross-validation to randomly create training (80%) and validation (20%) datasets from the overall training dataset with replacement and shuffling [66]. Optimal hyperparameters were chosen such that the validation score was maximized, and were subsequently used for prediction using the test dataset”*

Fourth, why including so many types of machine-learning methods but only selecting one or two to report the results? What are the standard of your modeling selections? Also, you mentioned the non-linearity as an advantage of machine-learning, but your study does not show any nonlinear analysis. It seems you mainly used the models for prediction, not about the relationships between variables, not about nonlinearity.

*As mentioned in the ‘Statistical Methods’ section (p. 15-16), we selected the SVR model because it provided the highest prediction accuracy among all the machine learning models we considered. Since bootstrap aggregation using SVR (i.e., the BAG model) did not provide significant improvements in prediction accuracy over SVR, we chose SVR as the most reliable model for subsequent analyses as it converges faster than BAG.*

*As per your suggestion, we have now only kept MLM and SVR model accuracy tests in the main body of the paper (updated Table 3) and moved the other machine learning models, which were tested but did not end up being used for the eventual predictive model, to Supp. Table 11. The Supplementary Materials section is now also uploaded as a separate file (rather than end of the manuscript previously), which appears to be a requirement by the journal.*

*Regarding non-linearity, we tested and confirmed that a number of variables were related to the outcome in a non-linear manner, which is important for achieving more accurate model predictions in our chosen SVR model. Additional in-depth examination of non-linear effects seems too tangential to the main purpose of our paper, but we have now added the plots illustrating non-linearity for each of the built environment predictors to Supp. Figure 2 and called these out on p. 16.*

*We also added the following to address your question about the standard for model selection to p.15 : “In order to address overfitting concerns related to machine learning models, we employed 10-fold cross-validation to randomly create training (80%) and validation (20%) datasets from the overall training dataset with replacement and shuffling [66]. Optimal hyperparameters were chosen such that the validation score was maximized, and were subsequently used for prediction using the test dataset”*

*We hope this clarifies and resolves your remaining concerns. We would appreciate if you could kindly let us know how you would like to proceed. In the interest of time, and given the approval of two reviewers so far, we hope an additional round of reviews will not be necessary. As you know, this paper has been in review for a long time, with an initial submission in November 2020. Our team has to be mindful about the publication schedule as one of the authors is going to be on the academic job market this fall and has been hoping to include the work in their research portfolio. We have sincerely tried to incorporate all the feedback we received so far and believe the paper makes an important contribution to the literature on urban pedestrian modeling and built-environment influences on pedestrian flows.*
